# Sleep loss as a cardiometabolic risk factor: a narrative review of clinical and public health implications

**DOI:** 10.1007/s44470-026-00144-1

**Published:** 2026-08-03

**Authors:** Firas K. Ghanem, Hrayr Attarian, Zeina Al-Khalil, Colette S. Kabrita

**Affiliations:** 1 Gilbert and Rose-Marie Chagoury School of Medicine, Lebanese American University (LAU), Byblos, Lebanon; 2 Department of Neurology, Center for Circadian and Sleep Disorders, Feinberg School of Medicine, Northwestern University, 633 N Saint Clair Street, Suite 5000, Chicago, IL 60611 USA; 3 Faculty of Medicine, American University of Beirut (AUB), Beirut, Lebanon; 4 Department of Sciences, Faculty of Natural and Applied Sciences, Notre Dame University-Louaize (NDU), Zouk Mikael, P.O. Box 72, Zouk Mosbeh, Lebanon

**Keywords:** Circadian misalignment, Sleep deprivation, Neuroendocrine regulation, Cardiometabolic disease, Metabolic syndrome, Autonomic dysfunction

## Abstract

**Purpose:**

To synthesize current evidence on how sleep deprivation, impaired sleep architecture, and circadian misalignment affect cardiovascular and metabolic regulation across autonomic, hormonal, inflammatory, and behavioral pathways, and to highlight remaining research gaps in cardiometabolic sleep medicine.

**Methods:**

A structured literature search was conducted using PubMed, Scopus, and Google Scholar to identify peer‑reviewed primary and review articles published within the past decade, using chronobiological and cardiometabolic search terms. Reference lists of retrieved articles were screened to identify additional relevant studies.

**Results:**

Experimental and epidemiologic data consistently link short or disturbed sleep with increased incidence of hypertension, coronary artery disease, stroke, obesity, type 2 diabetes, and metabolic syndrome. Sleep loss activates the sympathetic nervous system and elevates cortisol, while suppressing growth hormone, thereby promoting vasoconstriction, endothelial dysfunction, and cardiac remodeling. Parallel disruption of leptin, ghrelin, and endocannabinoid signaling increases hunger, caloric intake, and preference for energy‑dense foods, driving weight gain, visceral adiposity, and insulin resistance. Circadian misalignment from shift work and social jet lag further amplifies inflammatory signaling and metabolic risk across the lifespan. Limited interventional data suggest that sleep extension can improve appetite regulation, blood pressure, and some glycemic indices, but mechanistic and long‑term outcomes remain underexplored.

**Conclusion:**

Sleep deprivation and circadian disruption mediate cardiometabolic disease through converging neuroendocrine, autonomic, inflammatory, and behavioral pathways. Future research should employ longitudinal designs and integrated multi- “omics” approaches combined with sleep phenotyping to clarify causal mechanisms and identify novel biomarkers and therapeutic targets in clinical sleep medicine.

## Introduction

Sleep is fundamental to human health, and its disruption has been linked to a myriad of effects on the body. Both acute and chronic sleep deprivation adversely affects metabolism, immunity, mental health, and cardiovascular health [1]. Sleep quality is defined as adequate sleep duration and depth, sufficient to achieve the restorative effects of sleep that are integral to human health. Acute sleep deprivation and diseases like chronic insomnia and sleep-disordered breathing are associated with circadian misalignment and may interfere with the homeostasis achieved by adequate sleep quality and quantity [2].

Growing evidence consistently demonstrates strong associations between sleep disorders and several pathological conditions, including diabetes, coronary artery disease (CAD), hypertension, and cardiac arrhythmias [2, 3]. Evidence suggests a bidirectional relationship between sleep and cardiometabolic health, with current research focusing on the mechanisms underlying how sleep deprivation affects cardiovascular events [2]. A popular theory is that sleep deprivation is associated with inflammation, autonomic dysfunction, and hormonal imbalances, all of which are drivers of poor cardiovascular health [4–6]. For instance, the sympathetic arousal observed in sleep apnea has been directly linked to hypertension. Studies also show a “U-shaped” association between sleep duration and cardiovascular disease (CVD) risk, with both longer and shorter sleep durations than the normal range (6–9 h) conferring increased CVD risk [7, 8].

The link between sleep and health is not new. In the early days, simply observing that individuals with poor sleep schedules reported greater fatigue and reduced work efficiency supported the view that sleep is integral to proper physiological functioning. Today, this link is well established by studies that demonstrate the relationship between poor sleep quality and multisystem diseases [8]. In this narrative review, we aim to examine the effects of sleep disruption on cardiovascular health and metabolism. We conducted a comprehensive review of the literature examining the relationship between sleep and cardiovascular and metabolic diseases. The review further highlights key research considerations that remain unaddressed.

## Methods

**Fig. 1:**
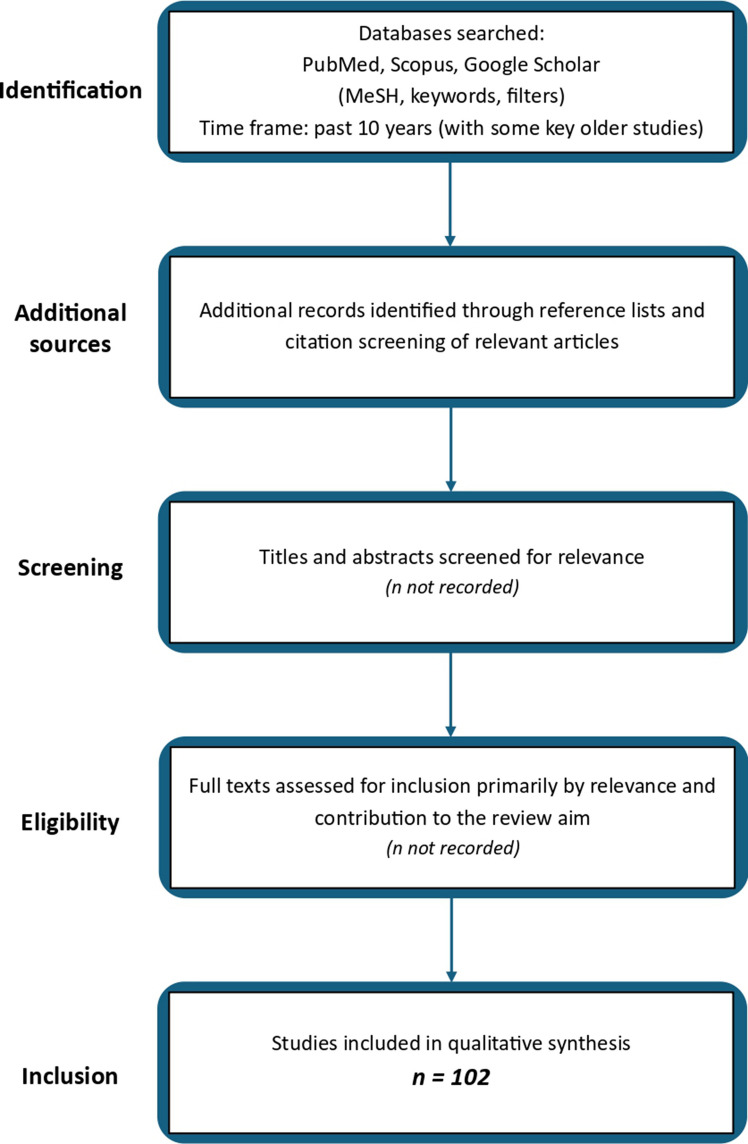
Structured literature selection process for this narrative review. This figure illustrates the stepwise process used to identify and select studies included in the narrative synthesis on sleep deprivation, circadian disruption, and cardiometabolic outcomes. Electronic databases (PubMed, Scopus, and Google Scholar) were searched using predefined chronobiology- and cardiometabolic-related terms. Additional studies were identified through reference list screening. Titles/abstracts and full texts were evaluated based on relevance to sleep–cardiometabolic mechanisms, methodological contribution, and inclusion of complementary or conflicting evidence. As this is a narrative review, intermediate screening counts were not prospectively recorded; therefore, only the final number of included studies (*n* = 102) is reported

This narrative review was conducted using a structured and iterative search of the literature to identify relevant studies linking sleep deprivation with cardiometabolic outcomes. Three electronic databases (PubMed, Scopus, and Google Scholar) were searched using combinations of chronobiology- and cardiometabolic-related keywords and MeSH terms, such as “circadian misalignment”, “sleep deprivation”, “sleep disorders”, “neuroendocrine regulation”, “energy homeostasis”, “insulin resistance”, and “cardiovascular disease”. Search results were screened by the authors to identify peer-reviewed articles, both primary research studies and review articles. Studies published within the last 10 years were prioritized, while some older studies were considered where necessary for mechanistic or epidemiological context. Only articles published in English were included. Additional relevant studies were identified through manual screening of reference lists of key retrieved articles. Titles and abstracts were screened for relevance to the review objective, followed by full-text assessment primarily based on relevance to sleep-cardiometabolic mechanisms, biological plausibility, and methodological contribution to understanding mechanistic or clinical associations between sleep disruption and cardiometabolic health. Where available, studies presenting complementary or conflicting findings were also included to enhance balance and reduce potential interpretive bias. A total of 102 studies were ultimately included in the final qualitative synthesis based on their relevance and contribution to the topic. Given the narrative design of this review, study identification and screening were not conducted using a fully predefined systematic protocol, and intermediate screening counts were not prospectively recorded. The overall literature search process is summarized in a structured flow diagram (Fig. 1).

This review aims to synthesize scattered findings linking cardiovascular morbidity to altered circadian neuroendocrine regulation following sleep disruption, while acknowledging the complexity and heterogeneity of the underlying literature. Narrative reviews are inherently susceptible to selection and interpretive bias; thus, limitations related to study heterogeneity, sex imbalance in mechanistic research, and potential bidirectional relationships between sleep and cardiometabolic disease are explicitly acknowledged in the Limitations section. A systematic review with predefined eligibility criteria and formal risk-of-bias assessment is warranted in future research to provide a more definitive synthesis of the evidence base.

## Results

### Sleep deprivation and cardiovascular disease

Chronic sleep deprivation is linked to cardiovascular health deterioration. This relationship has been consistently demonstrated and replicated across several studies, even after adjustment for other cardiac risk factors, including age, smoking, physical activity, weight, cholesterol, and diabetes. In a large longitudinal study spanning 7–25 years, Cappuccio et al. (2011) compiled evidence from over 474,000 participants across 8 countries. They found that habitual sleep duration of < 6 h per night was associated with a 48% higher risk of developing and/or dying from heart disease and a 15% increased chance of developing and/or dying from a stroke [9]. Studies of this kind showed that individuals with poorer sleep quality and/or duration (namely, sleeping < 6 h per night) were twice as likely to suffer from myocardial infarction (MI) or stroke during their lives as those sleeping 7–8 h on average. This association was particularly true when following up middle-aged adults (45 years or older). Optimizing sleep in middle age is thus crucial to a better, longer life. However, this is also the life stage when intense work and family obligations often lead adults to sacrifice sleep and neglect healthy sleep habits [10].

One way a lack of sleep disrupts the heart’s physiology is through blood pressure (BP) regulation. The association between short sleep and hypertension was demonstrated by a study that tracked individuals aged 30–90 years over 8–10 years. The researchers found that participants who habitually slept ≤ 5 h per night were significantly more likely to develop hypertension during the follow-up period than those who reported 7–8 h of sleep per night [11]. When unrecognized and untreated, hypertension substantially increases the risk of heart failure, CVD, stroke, chronic kidney disease, and other life-threatening complications. Globally, the silent epidemic of hypertension affects > 1 billion individuals and accounts for > 7 million deaths annually [12]. A cohort of 4810 subjects, 647 of whom had hypertension, was followed for 10 years. The adjusted hazard ratio (aHR) of hypertension in those who habitually slept ≤ 5 h per night was 2.10 (95% CI 1.58–2.79) [11]. Similarly, the Sleep Heart Health study reported increased risk of hypertension with < 7 h of sleep in their cohort of 2813 men and 3097 women. Subjects sleeping < 6 h and between 6 and 7 h per night had adjusted odds ratios (aORs) for hypertension of 1.66 (95% CI 1.35–2.04) and 1.19 (95% CI 1.02–1.39), respectively, compared with those sleeping 7–8 h [13]. Thus, insufficient sleep is a significant and modifiable risk factor contributing to the development and progression of hypertension, a growing public health burden [14].

Beyond hypertension, sleep deprivation exerts additional adverse effects on cardiovascular function, including elevations in heart rate (HR) and the development of tachycardia. Even sleep restriction by 1–2 h per night can measurably impair cardiovascular regulation. In a crossover study, a single night of acute sleep deprivation significantly increased 24-h systolic blood pressure by 5.2 mmHg and HR by 3.8 beats per minute [15]. Notably, these effects were observed in young, otherwise healthy individuals, highlighting the sensitivity of cardiovascular physiology to even acute sleep loss. In another study with 28 healthy volunteers (14 male and 14 female), total sleep deprivation (TSD) resulted in persistent tachycardia in response to an acute stressor without affecting either peripheral sympathetic activity or mean arterial pressure [16].

Sleep loss not only increases HR and BP but also interferes with the structural integrity of blood vessels, especially the coronary arteries. King et al. followed a cohort of 495 healthy subjects over 20 years. After adjusting for all variables, they reported that reducing sleep by 1 h only was associated with a 33% higher risk of coronary artery calcification [17].

Mechanistically, sleep deprivation compromises cardiovascular homeostasis in part through dysregulation of the sympathetic nervous system (SNS) [18]. Persistent SNS overactivity, as observed in sleep-restricted states, is associated with a pre-hypertensive and pro-atherogenic state marked by increased HR, vasoconstriction, endothelial dysfunction, and impaired vascular repair [18]. This shift toward autonomic imbalance represents a key mechanistic pathway linking sleep deprivation to cardiometabolic disease [18].

A substantial body of evidence indicates that insufficient sleep is consistently associated with heightened SNS activity [18]. Both acute and chronic sleep restriction induce a sustained state of autonomic imbalance that extends beyond the immediate period of sleep loss [19]. This phenomenon is particularly evident in individuals with chronic sleep curtailment due to occupational demands, sleep disorders, or behavioral factors (such as poor sleep hygiene) [20]. In addition, sleep deprivation–induced sympathetic overactivity is associated with sustained elevations in circulating cortisol levels. In healthy young men, compared to normal sleep duration, both adrenocorticotropic hormone (ACTH) and cortisol levels were elevated by 19% and 21%, respectively, following 2 nights of sleep restricted to 4 h [21]. In another cohort of healthy young women, sleep restriction to 3 h per night not only elevated afternoon and evening cortisol levels but also dampened the decline in cortisol throughout the day [22]. Chronic cortisol excess consequently is associated with vasoconstriction and contributes to the development and progression of hypertension, thereby increasing the risk of CVD [23]. While treating hypercortisolemia reduces CVD risk [23].

Sleep deprivation also suppresses growth hormone (GH) secretion [24]. GH is essential for repairing and maintaining the integrity of arterial endothelium [25]. In addition, GH plays an important role in maintaining myocardial mass. Hence, the increased risk of heart failure in GH deficiency [25]. Insufficient GH is associated with a higher likelihood of atherosclerosis and, thus, potentially myocardial or cerebral infarction, or stroke [26].

Collectively, these mechanisms illustrate how sleep deprivation initiates a cascade of physiological disturbances that adversely affect cardiovascular function. In contrast, adequate sleep, particularly during deep non–rapid eye movement stage 3 (N3) sleep, exerts a protective effect on cardiovascular homeostasis. During N3 sleep, parasympathetic activity increases and sympathetic activity, resulting in reductions in HR and BP. This sustained autonomic regulation mitigates physiological stress and lowers the risk of hypertension, MI, heart failure, and stroke [27]. Accordingly, deep N3 sleep can be considered a critical period for nocturnal cardiovascular recovery and regulation.

As a final illustration of the relationship between sleep and cardiovascular health, evidence from a large-scale “natural experiment” provides compelling insight. The transition to daylight saving time (DST), affecting > 1.5 billion individuals globally, results in an acute loss of ~ 1 h of sleep for a single night each year [28]. This population-level perturbation in sleep duration offers a unique opportunity to examine the short-term cardiovascular consequences of even modest sleep deprivation.

The DST experiment is an annual event in which clocks are adjusted by an hour, either forward or backward, resulting in people either losing or gaining an hour of sleep. In the Northern Hemisphere, when the clocks “spring forward” in March, many people miss out on an hour of sleep. By analyzing hospital records, Sadhu and colleagues found that this loss of sleep was associated with a significant, 24%, increase in MIs the day following the spring DST transition (RR1.24, 95% CI 1.05–1.46; *P* = 0.011) after adjustment for trend, seasonality, and hours of exposure. The opposite was also true. That is, in the fall, when the clocks “fall back” and people gain an hour of sleep, heart attack rates fell significantly by 21% the day following the fall transition (RR 0.79, 95% CI 0.62–0.99; *P* = 0.044) [28]. In contrast to these day-specific effects, no change was observed in the total weekly number of percutaneous coronary interventions (PCIs) for MI during the first week following DST transitions. These findings are consistent with earlier studies from Scandinavian, Croatian, and United States (US) cohorts reporting short-term increases in MI incidence following the spring DST transition [29–31], and are further supported by a review by Manfredini et al.’s review (2018) which analyzed of 87,944 DST-related MI events across six studies [32].

However, these findings should be interpreted with caution in the context of circadian cardiovascular physiology and underlying cellular mechanisms. Specifically, the temporal window required for synchronization between central and peripheral (including myocardial) clocks, along with associated neuroendocrine regulatory pathways, complicates direct causal interpretations following DST transitions. Moreover, the reported changes in MI incidence are modest and transient, derived primarily from observational studies lacking robust experimental validation in human populations. The heterogeneity across studies, including differences in geographic regions, cohort sizes, and methodologies, further limits generalizability. Consequently, the extent to which clock shifts such as DST meaningfully influence population-level MI incidence remains debated within the broader circadian cardiovascular literature [33, 34]. Additionally, more recent large-scale studies conducted in Ireland and the US reported no significant differences in MI incidence during DST transition weeks compared to adjacent weeks, nor any differences in in-hospital outcomes [35, 36]. These findings challenge the notion that the spring DST transition increases overall MI incidence.

Beyond cardiovascular outcomes, the effects of DST-related sleep disruption extend to other domains. Similar patterns have been observed in the number of traffic accidents, highlighting that the brain is just as sensitive as the heart to even small sleep disruptions [37]. Other studies involving DST reported a significant increase in death from suicide and substance abuse following the spring transition [38], while fatigue-induced cognitive bias was shown to influence judicial decisions [39], demonstrating the brain’s sensitivity to even minor doses of sleep disruption. While many people tend to think of losing an hour of sleep as unimportant, the effects are anything but trivial.

Taken together, circadian influences on cardiovascular physiology are well documented; however, many human datasets demonstrate modest effect sizes, context-dependent phase shifts, or near-null associations. Factors such as physical activity, comorbidities, dialysis status, healthcare system processes, and metabolic variability may obscure or override intrinsic circadian effects. As a result, the translation of mechanistic circadian insights into real-world cardiovascular outcomes remains incompletely understood.

### Sleep loss and metabolic syndrome

Sleep loss compromises appetite regulation and glucose metabolism. These impairments lead to weight gain, obesity, type 2 diabetes mellitus (T2DM), and metabolic syndrome [40, 41]. In addition to being a major risk for morbidity and mortality, diabetes is costly. In 2018, the American Diabetes Association estimated that diabetes-related health costs were $300 billion per year, and obesity-associated costs were $2 trillion [42].

Epidemiologically, multiple studies have reported an association between shorter sleep duration and T2DM. Irrespective of subject populations, cultural backgrounds, and geographic locations, these studies established a clear link between sleep loss and T2DM, even after adjusting for potential confounders [43–46]. Habitual short sleep duration is associated with increased ghrelin/leptin ratio (higher ghrelin and lower leptin). In a recent study, participants with short sleep duration had 14% higher ghrelin levels than those with normal sleep duration [47]. The endocannabinoid system, when stimulated, increases hunger and craving for high-calorie foods. Sleep restriction is associated with increased endocannabinoid levels [48]. As discussed in the context of cardiovascular regulation, sleep restriction also perturbs glucocorticoid (cortisol) secretion patterns. In sleep-deprived individuals, cortisol rises in the evening rather than in the morning [49]. In addition, its nadir levels are higher in sleep-deprived individuals than in normal sleepers, and its rate of decline is slower, leading to greater exposure to cortisol [49]. In addition to its cardiovascular effects, altered cortisol dynamics have important metabolic consequences, influencing glucose homeostasis, insulin sensitivity, and energy balance, which can lead to obesity. To establish the directionality and underlying mechanisms of this relationship, Van Cauter and colleagues enrolled 11 healthy young adult males and restricted their sleep to 4 h per night for 6 days. The participants were allowed 12 h of sleep per night for the following 6 nights [50]. Carbohydrate metabolism was assessed through an intravenous glucose tolerance test (ivGTT) and blood sample collections performed on the 5th and 6th days of each arm. There was a 40% drop in the glucose clearance rate during sleep restriction compared with extended sleep, indicating a prediabetic state [50]. The insulin response was also 30% lower in the sleep-restricted condition than during recovery sleep. Therefore, just 1 week of limited sleep was sufficient to trigger a metabolic dysfunction comparable to insulin resistance and T2DM [41–50]. A similar study with 38 women aged 20–75 demonstrated increased insulin resistance during a 6-week short-sleep period compared with a 6-week extended-sleep period [43]. Insulin resistance was independent of the degree of adiposity [43].

### Sleep problems and the development of obesity

Sleep restriction is strongly associated with increased body weight, in part through its effects on appetite regulation and energy homeostasis. Two key hormones, leptin and ghrelin, play antagonistic roles in fine-tuning appetite. Leptin, primarily secreted by white adipose tissue, signals satiety and suppresses food intake, whereas ghrelin, secreted mainly by the stomach, stimulates hunger [51]. Perturbations in the balance of these hormones can disrupt normal appetite regulation, promoting overeating and consequent weight gain. Sleep loss increases ghrelin and reduces leptin levels, thereby increasing caloric intake and promoting adiposity. These sleep loss–induced hormonal perturbations provide a mechanistic link between insufficient sleep and obesity risk [51]. In a trial with 19 healthy lean men, sleep restriction was associated with elevated ghrelin levels, particularly in the evening, and led to increased consumption of foods with high sugar content. This occurred despite leptin levels being unaffected [51]. Many other studies have also replicated these findings [52, 53]. Meanwhile, leptin levels drop with sleep deprivation. For instance, in a study of 44 young, healthy subjects (20 women and 24 men), sleep restriction resulted in significantly lower leptin levels and higher ghrelin levels [54].

There is ample evidence that suggests a concurrent decline in societal sleep duration with rising obesity rates [55], highlighting a potential behavioral contributor. A meta-analysis of several population-based studies demonstrated that habitual short sleep (≤ 6 h per night) increased the odds of obesity by 1.40 (95% CI 1.12–1.73) after adjusting for other variables [56].

In addition to weight gain, experimental sleep loss is associated with increased visceral abdominal adiposity. In a cohort of 12 healthy adults, sleep restriction to 4 h per night for 14 days resulted in a significant increase in abdominal fat, accompanied by higher protein and fat intake [57]. In another study with a larger cohort of 225 subjects (44% female), sleeping 4 h per night for 5 nights resulted in a 1-kg weight gain, compared with only 100 g of weight gain in the control group who slept 10 h per night. This was associated with increased caloric and fat intake in the sleep-restricted group [58]. Energy expenditure was not higher in the sleep-restricted group, but caloric consumption was, hence a primary reason for weight gain [59].

In addition to increased caloric intake, sleep deprivation tends to increase caloric intake from snacks and other “unhealthy” food choices. This is independent of changes in leptin and ghrelin levels. Nedeltcheva et al. assigned 11 healthy participants (5 women and 6 men) to either partial sleep deprivation (PSD) (5.5 h in bed) or a normal sleep condition (8.5 h in bed) for 2 weeks. The data from the PSD period was compared to the normal sleep period for each participant. Subjects had unrestricted access to food, and their daily caloric intake and physical activity were monitored. During the sleep-restricted period, average daily caloric intake increased by approximately 300 kcal relative to the normal sleep condition. In addition, more daily calories came from high-carbohydrate snacks in the PSD condition than in normal sleep conditions: 1087 ± 541 cal a day vs 866 ± 365 cal a day [60]. In a 2019 meta-analysis of 17 studies, Zhu et al. demonstrated that PSD resulted in 252.8 (95% CI, 59.1–446.5, *P* = 0.011) more daily calories consumed than under normal sleep conditions [61].

The sensation of hunger, or the desire to eat, is distinct from the actual behavior of overeating. This distinction raises the question of whether sleep deprivation is directly linked to increased food intake. In the same meta-analysis, Zhu et al. examined 15 studies that measured hunger on a visual analogue scale under both PSD and TSD conditions. Compared to normal sleep, subjective hunger increased by an average of 7.5 mm (95% CI 0.9–14.1, *P* = 0.027) with PSD, and, by an average of 22.8 mm (95% CI 9.7–35.9, *P* = 0.001) with TSD [61]. Broussard et al. in 2016 demonstrated similar findings regarding increased snack and carbohydrate consumption. They also linked it to an increase in evening ghrelin levels under PSD compared to normal sleep [51].

All this can impact weight. Zhu et al. in the same meta-analysis, examined weight changes across 10 studies, all using PSD. Over the duration of the study, ranging from 114 nights, the mean increase in weight with PSD was 0.34 kg (95% CI 0.11–0.57, *P* = 0.003) [61].

Early hypotheses proposed that hormonal alterations during sleep restriction might represent a normal adaptation to increased caloric demands associated with extended wakefulness, even under sedentary conditions [62]. However, this notion has been largely refuted. Several studies reviewed by Reutrakul and Van Cauter (2018), including that of Nedeltcheva et al. (2009), outlined previously, demonstrate that sleep curtailment has minimal or no effect on total energy expenditure [48, 60]. Even under conditions of TSD, energy expenditure increases by only ~ 134 kcal over 24 h compared with 8 h of sleep [63], and in some studies, these differences fail to reach statistical significance when compared to the same baseline of 8 sleep hours [64]. Thus, the long-held belief that sleep serves primarily as an energy-conserving process is no longer supported. In fact, many sleep-dependent physiological processes are metabolically demanding, with some exceeding the energy costs of wakefulness [65]. Importantly, the modest increase in energy expenditure during sleep loss is far outweighed by a concomitant increase in caloric intake and reductions in physical activity [48]. Collectively, this imbalance between higher caloric intake and lower energy expenditure represents another piece of the mechanistic puzzle linking chronic sleep deprivation to obesity.

Sleep deprivation not only increases total caloric intake but also alters food preferences. Across multiple studies, sleep-restricted individuals exhibit heightened cravings for energy-dense foods, particularly snacks with high sodium content (e.g., chips, popcorn, olives), high sugar content (e.g., cake, cookies, ice cream), and starchy carbohydrates (e.g., pasta, bread, potatoes). On average, these cravings increase by 30–40% relative to baseline, depending on individual preference. In contrast, intake of protein-rich foods, dairy, fruits, vegetables, and fatty foods increases only modestly, by approximately 10–15% following insufficient sleep [48, 66]. These findings further reinforce that sleep loss is strongly associated with the consumption of highly palatable, calorie-dense foods, thereby contributing to a positive energy balance and potential weight gain.

From a neurobiological perspective, sleep loss also perturbs the regulation of energy homeostasis by altering lipid-derived metabolites known as endocannabinoids. As their name implies, these endogenous ligands structurally resemble and functionally mimic exogenous cannabinoids, such as those found in recreational or medical cannabis, and similarly activate cannabinoid receptors, promoting increased appetite. Under normal conditions, endocannabinoid concentrations peak in the early afternoon, coinciding with habitual mealtimes, and decline to a minimum around midnight [67]. In 2016, Hanlon et al. randomised 14 healthy lean adults (11 men and 3 women) to 4 nights of normal sleep (up to 8.5 h) and 4 nights of sleep restricted to 4.5 h. When sleep-deprived, the circulating levels of the endocannabinoid 2-arachidonoylglycerol (2-AG) and its analog 2-oleoylglycerol (2-OG) declined more slowly in the evening and were higher near the end of the day than when subjects had normal sleep. They concomitantly assessed hunger, appetite, and food intake under controlled conditions. This insufficient-sleep-induced upregulation of the endocannabinoid system correlated with increased hedonic (snack-concentrated) food intake. This sleep deprivation–induced surge in circulating endocannabinoids, together with concomitant alterations in leptin and ghrelin, may synergistically underlie the overconsumption of calorie-dense foods, thereby increasing the risk of weight gain and obesity [67].

To elucidate the neural mechanisms underlying sleep loss–induced shifts in food preference. In 2013, Greer et al. enrolled 23 healthy, lean adults in a study with 2 experimental conditions separated by at least 1 week: a full-night sleep condition and a TSD condition. During each session, participants viewed 80 food images ranging from low- to high-calorie items, rating their desire for each item on a 1–4 scale while undergoing functional MRI scanning [68]. Following the scan, participants were provided with a serving of the food they rated highest in desire, ensuring that the ratings reflected genuine appetitive motivation rather than experimental demand characteristics [68]. This design allowed the investigators to directly link neural activity patterns to self-reported food craving under well-rested and sleep-deprived states [68]. Hence, using within-subject comparisons of brain activity across sleep conditions provided robust evidence for the neural mechanisms linking sleep deprivation to altered eating behavior. Specifically, sleep loss increased amygdala activation, a key limbic structure involved in emotional and reward processing, in response to images of palatable, energy-dense foods. Concurrently, activity in the prefrontal cortex, which mediates executive control and decision-making, was attenuated. This functional imbalance (heightened hedonic signaling coupled with impaired impulse control) was associated with a significant shift in food preferences toward high-calorie items. Quantitatively, sleep-deprived participants exhibited an average increase of ~ 600 kcal in desired food intake. In contrast, sufficient sleep restored functional connectivity between the prefrontal cortex and subcortical reward centers, facilitating top-down regulation of hedonic drives and supporting behavioral restraint in food consumption [69]. These findings add a neural dimension to the mechanistic picture, highlighting how sleep deprivation interacts with hormonal and behavioral pathways resulting in hyperphagia and weight gain.

Beyond hormonal and neurocognitive alterations, recent evidence highlights a link between sleep loss and dysbiosis of the gut microbiome. The intestinal microbiome (a complex ecosystem of bacteria, fungi, protozoa, and viruses that affect the enteric nervous system, or ENS) plays a central role in maintaining overall gut metabolic and immune homeostasis. Dominant bacterial phyla include Bacteroidetes and Firmicutes, whose relative abundance is typically balanced in healthy individuals [70]. Sleep disruption has been shown to perturb this balance, decreasing Bacteroidetes while increasing Firmicutes [71]. Notably, alterations in the Bacteroidetes-to-Firmicutes ratio have been associated with weight gain and obesity in both animal and human models [72]. They may predispose individuals to insulin resistance and T2DM under chronic conditions. Mechanistically, elevated cortisol (a hallmark of stress-induced sympathetic activation during sleep deprivation) is associated with the proliferation of pathogenic bacteria [73], thereby contributing to a spectrum of gastrointestinal and metabolic disturbances [74].

Lastly, circadian misalignment can lead to weight gain and an increase in prevalence of obesity and metabolic syndrome. Shift work, for instance, is associated with increased proinflammatory and a decline in anti-inflammatory proteins. In a group of 15 healthy male shiftworkers, 24-h tumor necrosis factor alpha and interleukin 6 levels were higher than those in the control group of 7-day workers. In addition, adiponectin, an anti-inflammatory protein that regulates metabolism and reduces insulin resistance, was lower in the shift work group suggesting a mechanism by which circadian disruption can lead to metabolic syndrome and obesity [75]. Similarly, cortisol levels and insulin resistance were higher in the shift work group [76].

Social jet lag, or a large variation between sleep timing and chronotype that results in sleep deprivation, is associated with higher prevalence of obesity. In a cohort of 815 community-dwelling adults (605 non-obese, 100 obese without comorbidities, and 85 obese with metabolic syndrome), individuals with obesity and metabolic comorbidities exhibited significantly greater social jet lag, measured in hours, than the other 2 groups [77]. Across the cohort, increasing degrees of social jet lag (0–3 h) were associated with progressive elevations in inflammatory and metabolic markers, including C-reactive protein and glycated hemoglobin (HbA1c) [77]. Moreover, greater social jet lag (in hours) was associated with higher body mass index (BMI), greater fat mass (in kilograms), and larger waist circumference [77].

Roenneberg et al. queried their chronotype database of 65,000 unique entries and found that BMI increased commensurate with the magnitude of social jet lag and reduction in sleep duration during the workweek [78]. Mota and her colleagues collected social jet lag and nutritional data from 792 individuals (73% women with a mean age 55.9 ± 12.4 years). Social jet lag was associated with higher caloric intake, particularly after 9 PM, as well as evening snacking and higher fat consumption [79].

These associations extend to pediatric populations. In a study of 31 overweight and obese adolescents (77% female), Simon and colleagues demonstrated that greater social jet lag was associated with reduced insulin sensitivity. The authors also reported shorter weekday sleep duration among individuals with an evening chronotype, as well as a positive association between insulin resistance and a wider phase angle between bedtime and melatonin onset, indicative of delayed sleep–wake phase [80]. In another cohort of 59 obese adolescent girls with polycystic ovary syndrome (PCOS), the same group identified a significant association between morning circadian misalignment and insulin resistance, compared with 33 age-matched obese controls without PCOS. Morning circadian misalignment was quantified as the interval between melatonin offset time and clock time. Notably, all participants were screened for sleep-disordered breathing, which was excluded as a confounding factor [81].

Extending these observations to earlier developmental stages, the impact of sleep deprivation is particularly pronounced in early life, a critical period for metabolic programming and long-term health trajectories. Epidemiological evidence indicates that insufficient sleep in childhood is strongly associated with increased obesity risk. For instance, Guo et al. (2021) reported a 35–41% higher likelihood of obesity among Chinese children and adolescents aged 6–17 years with shorter sleep durations [82]. Notably, this relationship emerges even earlier in life; Lumeng et al. (2007) demonstrated a similar association in children as young as 2–7 years, underscoring the early onset of this risk [83]. These findings raise important concerns regarding behavioral and environmental determinants of sleep in pediatric populations. Inadequate prioritization of sleep by caregivers may inadvertently foster unhealthy lifestyle patterns from a young age, predisposing children to sustained metabolic dysregulation. Given the well-established interplay among sleep, neuroendocrine regulation, and energy balance, early-life sleep curtailment may represent a critical, modifiable risk factor that contributes to the lifelong burden of obesity and its associated cardiometabolic complications [84].

In addition to its effects on glucose metabolism and hormonal regulation, sleep deprivation also impairs fat utilization. In 2010, Nedeltcheva et al. examined the impact of sleep restriction on the efficacy of a hypocaloric diet among overweight individuals seeking a leaner body appearance [85]. Participants were assigned to 2 sleep conditions (5.5 h vs. 8.5 h in bed) for 2 weeks each, with a washout period in between. Although comparable weight loss was observed under both conditions, the composition of weight loss differed markedly. Under adequate sleep conditions, > 50% of the weight loss was attributable to reduction in adipose tissue. In contrast, during sleep restriction, over 60% of weight loss was attributable to lean tissue rather than fat [85]. These findings suggest that insufficient sleep impairs fat oxidation and shifts energy utilization away from lipid stores, thereby promoting the preservation of adipose tissue at the expense of lean body mass. Consequently, sleep deprivation may compromise the metabolic benefits of caloric restriction and hinder improvements in body composition.

Taken together, the previous findings indicate that the global obesity epidemic cannot be attributed to a single causal pathway, and sleep deprivation is no exception. While increased food consumption, larger meal portions, elevated caloric intake, and reduced physical activity remain key contributors, these factors alone are insufficient to account for the current magnitude of obesity prevalence [86]. Rather, a broader, multifactorial framework is required. In this context, a substantial and growing body of evidence has established a robust association between sleep curtailment and obesity risk. This relationship has been consistently demonstrated across epidemiological studies and contributes to the parallel rise of two major public health challenges, with significant implications for morbidity and mortality. Notably, longitudinal population data over the past 5 decades reveal a striking temporal convergence whereby declining sleep duration mirrors the upward trajectory of obesity rates. Such synchronized trends further reinforce the role of insufficient sleep as a critical yet often underappreciated determinant within the complex pathophysiology of obesity.

From a systems perspective, insufficient sleep, now prevalent in modern populations, induces a coordinated disruption of endocrine, neural, and behavioral pathways governing energy balance. Alterations in key appetite-regulating hormones (namely leptin, ghrelin, and endocannabinoids) drive increased hunger and caloric intake, while concurrent impairments in reward processing and executive control bias food selection toward energy-dense options. These effects, coupled with microbiome alterations and reduced responsiveness to dietary interventions, are related to a sustained positive energy balance despite relatively unchanged energy expenditure. Over time, this integrated metabolic dysregulation contributes to the development of obesity, T2DM, and their associated morbidity and mortality.

### Sleep loss and cardiometabolic disease

As highlighted above, short sleep and sleep deprivation are risk factors for metabolic disease, diabetes, and CVD. In a 2021 meta-analysis of 36 studies, Hua and colleagues demonstrated that habitual short sleep (defined as ≤ 6 h) in cohort studies was significantly associated with higher incidence of metabolic syndrome with a risk ratio (RR) of 1.15 (95% CI 1.05–1.25, *P* < 0.001). In cross sectional studies, it was also associated with higher prevalence of metabolic syndrome with an odds ratio (OR) of 1.06 (95% CI 1.01–1.11, *P* < 0.001) [87].

Short sleep defined as < 7 h was also associated with higher cardiovascular mortality in another meta-analysis of 19 studies with a RR of 1.19 (95% CI 1.13–1.26, *P* < 0.001) [88]. It has also been long established that metabolic syndrome increases the risk of cardiovascular morbidity and mortality. In a landmark study of 4483 subjects of 35–70 years of age, Isomaa et al. demonstrated that metabolic disease significantly increased the risk of CAD, MI, and stroke. The RRs were 2.96, 2.63, and 2.27 respectively (p 0.001) [89].

It has also been demonstrated that short sleep increases the risk of T2DM. People with habitual short sleep duration (< 4.5–6 h per night) had significantly higher levels of HbA1c (mean difference: 0.23%; 95% CI, 0.10–0.36) compared to normal sleep duration (6–8 h per night) [90]. Lastly, obesity is also associated with short sleep duration of ≤ 6 h. Short sleepers had a higher BMI (1.1 ± 0.36 kg, m(−2), *P* < 0.05) and fat mass (difference: 2.4 ± 0.64 kg, *P* < 0.05) over a period of 6-years [83]. Interestingly those subjects who increased their sleep from ≤ 6 to 7–8 h significantly decreased their BMI and fat mass [91]. T2DM is of course a risk factor for CVD. Risk of heart failure after adjusting for age remained high (OR, 2.8, 95% CI 2.2–3.6). Cardiovascular death rate is 4.4-fold higher in T2DM and twofold higher in stroke [92]. Obesity was also associated with an increased risk of CVD (aHR 1.36, 95% CI 1.10–1.67) and stroke (aHR 1.40, 95% CI 1.02–1.92) [93]. In addition to the above interventional study that showed a decrease in body weight with increasing sleep duration [91], there are a few other interventional studies. Tasali et al. reported a 14% decline in appetite and a 65% decline in craving for unhealthy snacks in 10 subjects after extending their sleep from 6.5 to 8.5 h per night over a 2-week period [94]. Al Khatib and colleagues demonstrated a 9.6 g reduction in sugar consumption and an average increase of 21 min in sleep per night over 4 weeks [95]. Haack et al. however, did not demonstrate a change in caloric intake, with an average 35-min increase in sleep per night over 6 weeks. Nevertheless, BP significantly improved with sleep extension [96]. Rachel Leproult et al. studied 13 women and 3 men who were all healthy and non-obese with a mean age of 25. The subjects were instructed to keep habitual sleep duration in the first 2 weeks and followed by 6 weeks of increased sleep duration by an hour per night. Objectively the mean sleep time increased by 44 min. Although there was no significant change in overall insulin and glucose levels before and after the intervention, the percent changes in fasting glucose and insulin were more pronounced with sleep extension, showing a positive correlation for glucose (*r* =  + 0.53, *P* = 0.041) and a negative correlation for insulin levels (*r* =  − 0.60, *P* = 0.025). Additionally, insulin sensitivity improved significantly with sleep extension (*r* =  + 0.76, *P* = 0.002), indicating that increasing sleep duration can enhance insulin sensitivity in adults who are habitually sleep restricted [97]. This suggests that even modest increases in sleep duration may positively influence glucose metabolism and reduce cardiometabolic risk by improving insulin action.

Taken together, current evidence suggests that sleep loss is not simply an isolated risk factor but a systemic cardiometabolic stressor. Insufficient sleep disrupts neuroendocrine, autonomic, and circadian regulation, promoting sympathetic activation, elevated cortisol, insulin resistance, and weight gain; in this setting, metabolic and vascular dysfunction interact to accelerate cardiovascular risk. Circadian misalignment across peripheral clocks in metabolic and cardiovascular tissues further amplifies inflammation and impairs vascular repair. This perspective supports the need for approaches that address sleep, circadian timing and alignment among biological clocks, and cardiometabolic health as interconnected processes rather than discrete domains.

## Discussion

### Study limitations

While this review provides a comprehensive synthesis of current evidence, several limitations inherent to its narrative design should be considered when interpreting the present findings. First, study selection was not conducted using a formal systematic protocol such as PRISMA guidelines, introducing potential selection bias. Despite the use of a structured search strategy and inclusion of studies with differing and conflicting findings where available, the possibility of preferential inclusion of certain lines of evidence cannot be entirely excluded. Second, the evidence base is highly heterogeneous, across study designs, populations, and methodologies. Included studies range from small, controlled crossover studies to large observational cohorts with variability in sample size, duration of follow-up, sleep assessment methods (each with inherent limitations), and outcome definitions. This limits direct comparability and precludes quantitative synthesis. Third, much of the literature is observational, restricting causal inference and leaving open the possibility of reverse causality, whereby underlying cardiometabolic conditions may also influence sleep. Fourth, generalizability may be limited by variation in study populations, including differences in age, comorbidities, occupational exposures (e.g., shift work), and geographic regions. Notably, several mechanistic studies have been conducted predominantly in male participants, which may limit applicability to female populations given known sex differences in sleep architecture, hormonal regulation, and cardiometabolic responses. Fifth, publication bias and selective reporting cannot be excluded, as studies demonstrating significant associations are more likely to be published. Additionally, the inclusion of both primary studies and secondary reviews may introduce overlap in reported findings, although care was taken to prioritize original data where possible. Finally, the rapidly evolving nature of sleep and circadian research means that emerging data may further refine or challenge existing conclusions. Future research employing systematic review methodologies with predefined inclusion criteria, formal risk-of-bias assessment, and quantitative meta-analysis is warranted to provide a more objective and definitive evaluation of the relationship between sleep disruption and cardiometabolic health.

### Conclusion and future directions

**Fig. 2:**
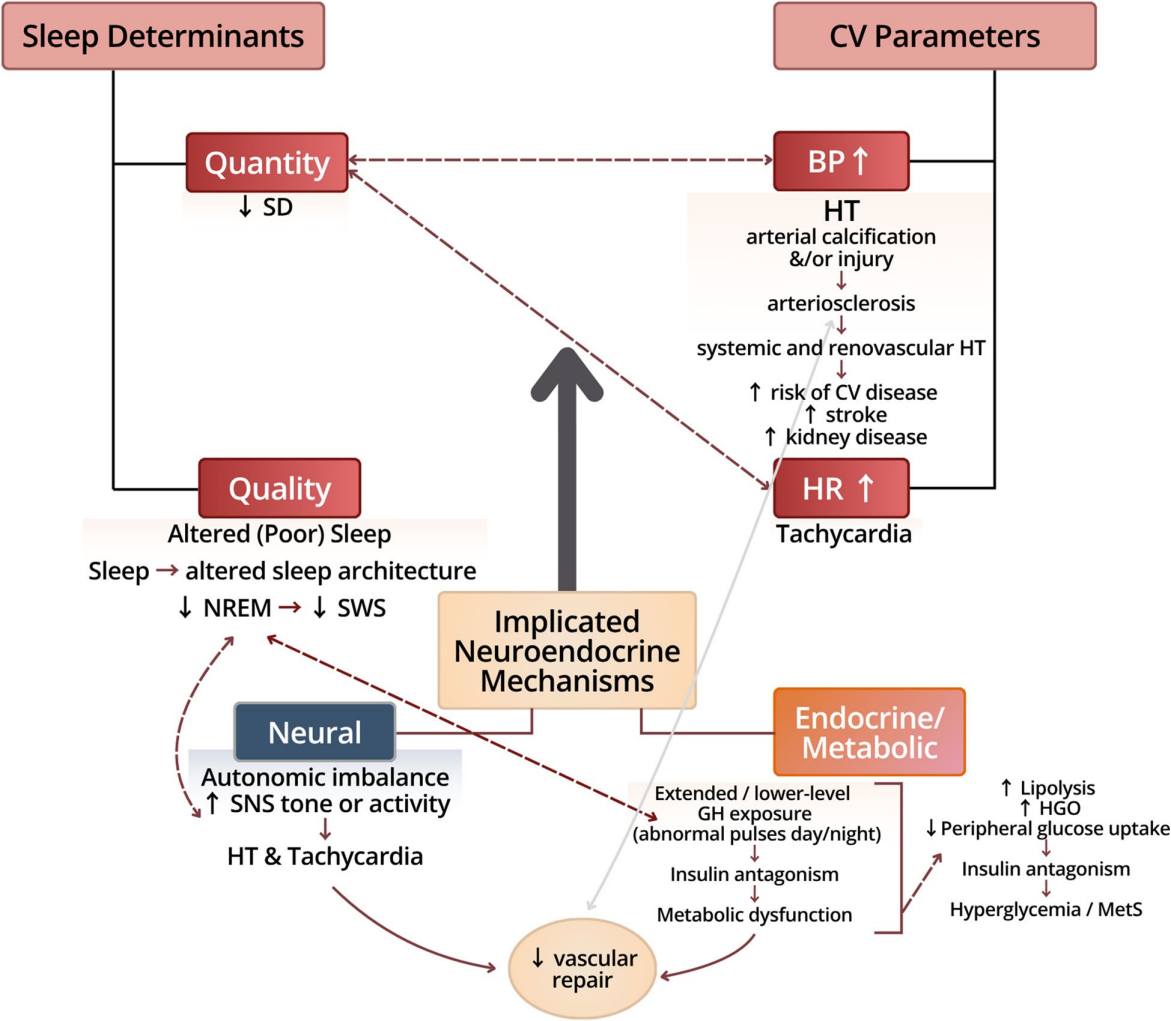
Schematic representation of sleep-cardiovascular interactions mediated by neuroendocrine mechanisms. Reduced sleep duration and poor sleep quality, particularly decreased N3 (slow-wave sleep), contribute to autonomic imbalance characterized by increased sympathetic nervous system activity, leading to hypertension and tachycardia. Concurrently, endocrine and metabolic alterations, including elevated cortisol levels, reduced growth hormone secretion, and insulin resistance with impaired glucose metabolism, are associated with metabolic dysfunction and hyperglycemia. These processes converge to induce endothelial dysfunction, impair vascular repair mechanisms, and are associated with athero-/arterio-sclerosis and myocardial remodeling. Collectively, these effects increase the risk and morbidity associated with systemic and renovascular hypertension, stroke, chronic kidney disease, and other cardiovascular complications. *BP*, blood pressure; *CV*, cardiovascular; *GH*, growth hormone; *HGO*, hepatic glucose output; *HR*, heart rate; *HT*, hypertension; *MetS*, metabolic syndrome; *SD*, sleep duration; *SNS*, sympathetic nervous system

Sleep is recognized as a fundamental regulator of cardiometabolic health rather than a passive state of physiological inactivity. The accumulating body of evidence reviewed here indicates that disturbances in sleep duration, sleep architecture, and circadian organization profoundly affect the neuroendocrine systems that govern cardiovascular and metabolic homeostasis (as shown in Fig. 2). Experimental and epidemiological studies consistently demonstrate that sleep deprivation disrupts key hormonal pathways (including the somatotropic axis, appetite-regulating signals such as leptin and ghrelin, and insulin-mediated glucose regulation), thereby promoting insulin resistance, obesity, hypertension, and other cardiometabolic disorders [98, 99]. At the systems level, these alterations converge with sympathetic overactivation, endothelial dysfunction, and inflammatory signaling to create a biological milieu that favors the development and progression of CVD.

Importantly, the relationship between sleep and cardiometabolic physiology is highly multidimensional. Sleep architecture (particularly the quantity and integrity of N3 sleep) plays a critical role in metabolic regulation, influencing growth hormone secretion, glucose utilization, and autonomic balance. Concurrently, circadian misalignment can disrupt the temporal synchronization of peripheral molecular clocks, including those within cardiomyocytes and metabolic tissues, thereby amplifying cardiometabolic vulnerability. These insights highlight the need to conceptualize sleep not merely in terms of duration but as a complex, multi-layered biological process that integrates neuroendocrine signaling, circadian timing, and metabolic regulation [100].

Despite substantial progress in delineating these mechanisms, significant knowledge gaps remain (as summarized in Table 1). Much of the current evidence derives from short-term laboratory studies or cross-sectional observational cohorts, which often lack comprehensive hormonal profiling or integrative molecular analyses. Critical endocrine pathways (including growth hormone pulsatility, the adipo-insular axis, and circadian hormone dynamics) remain incompletely characterized in the context of chronic sleep disruption. Furthermore, relatively few studies have integrated genomic, transcriptomic, and/or metabolomic data with detailed sleep phenotyping, limiting our understanding of the molecular networks through which sleep disturbances influence cardiometabolic disease risk. Addressing these gaps will require coordinated research efforts that incorporate standardized sleep measurement techniques, diverse longitudinal cohorts, and multi-omics approaches capable of capturing the complex physiological landscape linking sleep to cardiometabolic regulation [101, 102].

**Table 1:** Key knowledge gaps linking sleep disruption to cardiometabolic disease and priority directions for future research

Current knowledge gap	Limitations in existing evidence	Priority research directions
Heterogeneity in sleep assessment	Many studies rely on self-reported sleep duration or single-night recordings, limiting the reliability of sleep architecture characterization	Standardize objective sleep measurements using multi-night polysomnography, actigraphy, and spectral analysis of SWS to improve reproducibility across studies
Incomplete characterization of endocrine dynamics	Most studies measure static hormone concentrations rather than circadian rhythmicity or pulsatile secretion patterns	Implement high-resolution circadian hormone sampling to characterize diurnal dynamics of growth hormone, cortisol, insulin, leptin, and ghrelin in sleep-restricted conditions
Limited causal inference	A large proportion of current evidence derives from cross-sectional or short-term laboratory studies linking sleep loss to metabolic dysfunction	Conduct longitudinal cohort studies, mechanistic experiments, and Mendelian randomization analyses to clarify causal relationships between sleep disturbances and cardiometabolic outcomes
Underrepresentation of diverse populations	Many sleep-metabolism studies focus on relatively homogeneous cohorts from high-income countries	Expand recruitment to diverse populations across age, sex, ethnicity, and socioeconomic backgrounds to evaluate the generalizability of sleep–cardiometabolic relationships
Deficit in multi-omics integration	Few investigations combine sleep phenotyping with genomic, transcriptomic, proteomic, or metabolomic profiling	Integrate multi-omics approaches with sleep studies to identify molecular pathways linking circadian disruption, endocrine signaling, and cardiometabolic disease risk
Circadian misalignment and peripheral clock dysfunction	The interaction between sleep restriction and desynchronization of peripheral molecular clocks remains incompletely understood	Investigate temporal synchronization of peripheral clocks—including cardiomyocyte and metabolic tissue clocks—under conditions of sleep loss and circadian disruption
Cardiovascular structural and vascular effects	Mechanistic data on myocardial remodeling, endothelial repair, and vascular inflammation in response to sleep disruption remain limited	Use imaging, vascular biomarkers, and translational models to evaluate how chronic sleep loss contributes to endothelial dysfunction, hypertension, and structural cardiac changes
Limited interventional trials	Few randomized trials evaluate whether improving sleep directly reverses cardiometabolic dysfunction	Conduct randomized clinical trials of sleep-targeted interventions (sleep extension, CBT-I, CPAP for obstructive sleep apnea) with cardiometabolic and endocrine endpoints

Future research should also prioritize mechanistic and translational investigations to identify actionable therapeutic targets. Longitudinal cohort studies, Mendelian randomization analyses, and deeply phenotyped experimental models will be essential for disentangling causal pathways linking sleep disruption to cardiometabolic pathology. Equally important is the development of interventional trials evaluating whether sleep-focused therapies (including sleep extension, cognitive behavioral therapy for insomnia, or treatment of sleep-disordered breathing) can restore hormonal homeostasis and improve cardiometabolic outcomes. Such trials should incorporate detailed endocrine and metabolic endpoints, including diurnal hormone rhythms, insulin sensitivity, autonomic function, and vascular biomarkers.

Ultimately, advancing this field will require a shift toward integrative, systems-level research frameworks that bridge sleep physiology, circadian biology, endocrinology, and cardiovascular science. By elucidating the multi-omics architecture of sleep-dependent metabolic regulation, future investigations may uncover novel biomarkers and therapeutic strategies to mitigate the growing global burden of cardiometabolic disease. As modern lifestyles continue to erode the quantity and quality of sleep worldwide, understanding (and harnessing) the biological restorative functions of sleep represents a critical frontier in preventive medicine and population health.

## Data Availability

Our manuscript has no associated data.

## Abbreviations

*2-AG*: 2-Arachidonoylglycerol
*2-OG*: 2-Oleoylglycerol
*ACTH*: Adrenocorticotropic hormone
*aHR*: Adjusted hazard ratio
*aOR*: Adjusted odds ratios
*BMI*: Body mass index
*BP*: Blood pressure
*CAD*: Coronary artery disease
*CVD*: Cardiovascular disease
*DST*: Daylight saving time
*ENS*: Enteric nervous system
*GH*: Growth hormone
*HR*: Heart rate
*HbA1c*: Hemoglobin A1c
*ivGTT*: Intravenous glucose tolerance test
*MeSH*: Medical subject headings
*N3 sleep*: Non-rapid eye movement stage 3 sleep
*MI*: Myocardial infarction
*OR*: Odds ratio
*PCOS*: Polycystic ovary syndrome
*PSD*: Partial sleep deprivation
*RR*: Risk ratio
*SNS*: Sympathetic nervous system
*T2DM*: Type 2 diabetes mellitus
*TSD*: Total sleep deprivation
